# Ixekizumab‐induced urticarial drug eruption

**DOI:** 10.1002/ski2.271

**Published:** 2023-07-13

**Authors:** Ayaka Kaneoka, Natsuko Saito‐Sasaki, Etsuko Okada, Yu Sawada

**Affiliations:** ^1^ Department of Dermatology University of Occupational and Environmental Health Kitakyushu Japan

## Abstract

Biological agents targeting inflammatory skin diseases have dramatically overcome many of the limitations of older oral therapeutic options. Among the various biological agents, ixekizumab is a humanised monoclonal antibody that blocks the biological activity of IL‐17A, which exhibited high efficacy against psoriasis. Although there are a limited number of cutaneous adverse reactions, biologic‐induced type I allergic reactions are rare. Herein, we report a case of ixekizumab‐induced urticaria.

## Graphical abstract

This case report presents a drug‐induced urticaria as a unique clinical manifestation caused by ixekizumab.

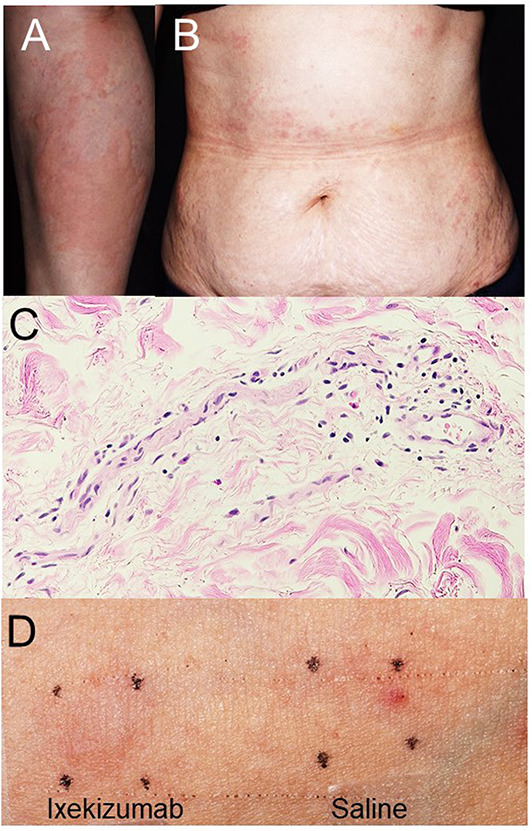

## 1 CASE REPORT

A 51‐year‐old woman had palmoplantar pustulosis since the age of 16 years. She experienced joint pain in her fingers, and adalimumab was administered for palmoplantar pustulosis‐associated arthritis; however, it was discontinued due to an anaphylactic reaction. Instead of adalimumab, she was treated with methotrexate and secukinumab, which were ineffective for arthritis. In addition, there were no additional therapeutic options at that time; ixekizumab was administered off‐label for palmoplantar pustulosis by internal department doctors in our hospital after obtaining informed consent from the patient. Three months after ixekizumab administration, the development of urticaria was observed repeatedly within several hours. She was referred to our department for skin eruption examination. The patient was treated with methotrexate, foraminiferan, lansoprazole, and biotin.^1^, ^2^

**FIGURE 1:**
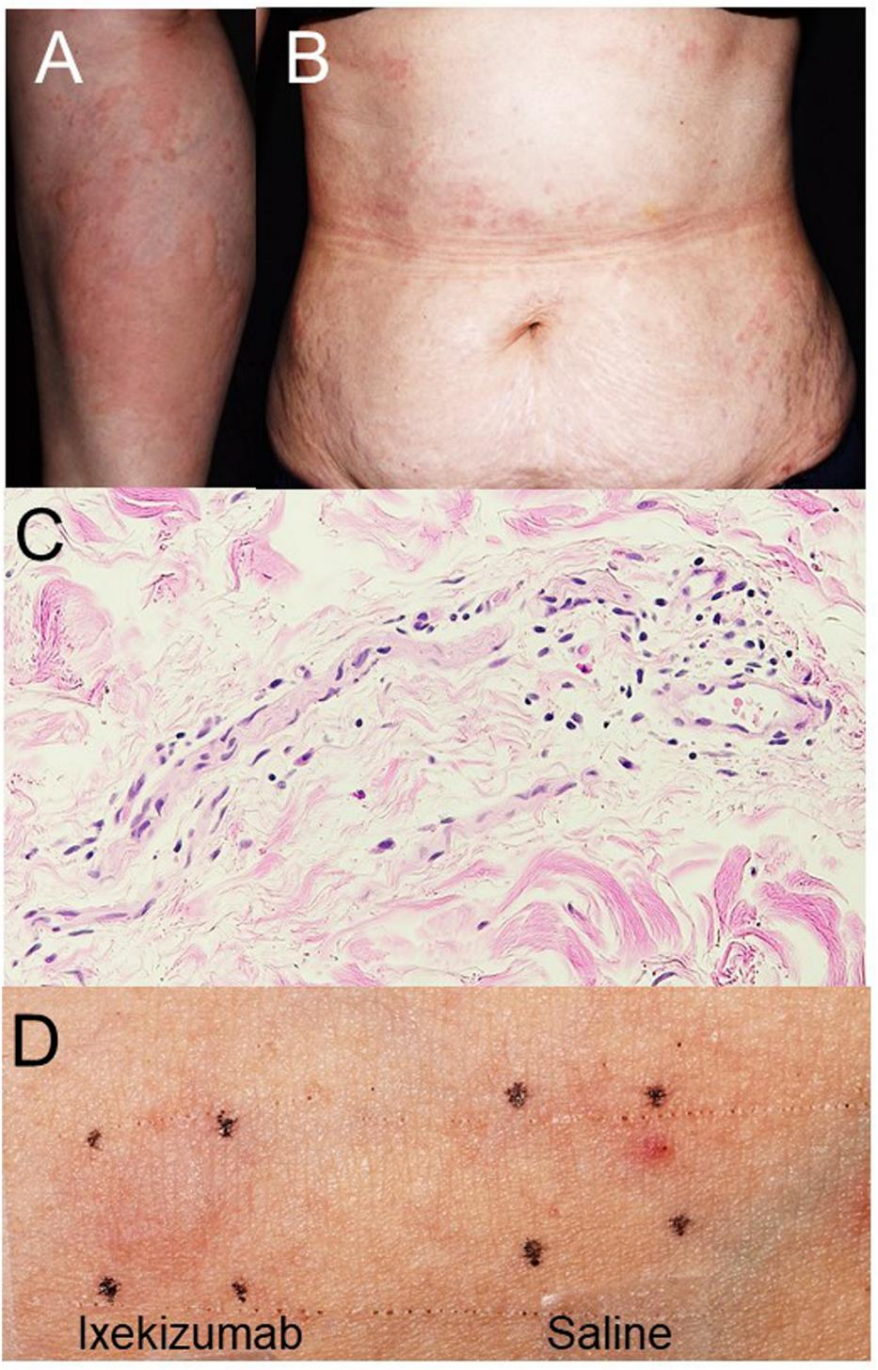
Clinical manifestation, histological analysis, and skin test. (a) Clinical manifestation. Erythematous wheal was observed in whole her body. (b) Histological examination. Eosinophils and mast cells were infiltrated into the dermis. (c) The intradermal skin test. The intradermal injection test showed a positive response 10 min after ixekizumab administration.

Physical examination revealed an erythematous wheal that had developed throughout his body (Figure 1a). A skin biopsy showed infiltration of inflammatory cells, such as eosinophils and mast cells, into the superficial dermis (Figure 1b). An intradermal injection test revealed a positive reaction 10 min after ixekizumab administration (Figure 1c). Therefore, we diagnosed the skin eruption as an urticarial reaction to ixekizumab.

## 2 DISCUSSION

We speculated that there are three possible causes of urticaria following ixekizumab treatment. The first possibility is the mechanistic action of IL‐17 blockade. However, IL‐17 is involved in the pathogenesis of various types of urticaria.^3^ Therefore, ixekizumab‐mediated IL‐17 blockade may de‐escalate the severity of urticaria. The second possibility is additive urticaria. Ixekizumab contains several additives, such as polysorbate 80, a representative agent that causes type I allergy.^4^ Indeed, two previously reported cases of polysorbates 80 and 20 were related to urticaria (Supplementary Table 1). Our patient had a history of anaphylaxis after adalimumab administration. Adalimumab and ixekizumab contain 80. However, our patient did not develop urticaria or anaphylaxis following the first administration of ixekizumab, and prick tests for polysorbates 20 and 80 showed negative results, indicating that polysorbate 80 might be less likely to trigger urticarial drug reactions. The third possibility is the presence of a specific type I allergic reaction to ixekizumab. To examine the presence of the same cases related to biologic‐induced urticaria, we summarised urticarial drug eruptions following biologics (Supplementary Table 2). There were four cases of biologic‐related urticaria. Three of the four patients were women. Three patients developed urticaria after adalimumab administration, and our report is the first case of IL‐17 blockage‐mediated urticaria. Therefore, it should be considered that other biological agents, not limited to TNF inhibitors, are also involved in triggering urticarial drug eruption.

## CONFLICT OF INTEREST STATEMENT

The authors declare no conflicts of interest.

## AUTHOR CONTRIBUTIONS

**Ayaka Kaneoka**: Data curation (Lead); Formal analysis (Lead); Investigation (Lead). **Natsuko Saito‐Sasaki**: Data curation (Lead); Formal analysis (Lead); Investigation (Lead). **Etsuko Okada**: Conceptualisation (Equal); Formal analysis (Equal). **Yu Sawada**: Conceptualisation (Lead); Investigation (Supporting); Project administration (Lead).

## ETHICS STATEMENT

Not applicable.

## Supporting information

Supplementary Material

## Data Availability

Data sharing is not applicable to this article as no new data were created or analyzed in this study.

## References

[1] Saeki H , Terui T , Morita A , Sano S , Imafuku S , Asahina A , et al. Japanese guidance for use of biologics for psoriasis (the 2019 version). J Dermatol. 2020;47(3):201–222. 10.1111/1346-8138.15196 31916326

[2] Rodríguez‐Jiménez B , Domínguez‐Ortega J , González‐Herrada C , Kindelan‐Recarte C , Loribo‐Bueno P , Garrido‐Peño N . Successful adalimumab desensitization after generalized urticaria and rhinitis. J Investig Allergol Clin Immunol. 2009;19(3):246–247. 19610276

[3] Sabag DA , Matanes L , Bejar J , Sheffer H , Barzilai A , Church MK , et al. Interleukin‐17 is a potential player and treatment target in severe chronic spontaneous urticaria. Clin Exp Allergy. 2020;50(7):799–804. 10.1111/cea.13616 32412136

[4] Pérez‐Pérez L , García‐Gavín J , Piñeiro B , Zulaica A . Biologic‐induced urticaria due to polysorbate 80: usefulness of prick test. Br J Dermatol. 2011;164(5):1119–1120. 10.1111/j.1365-2133.2011.10220.x 21219296

