# Supplementary material for: Ixekizumab‐induced urticarial drug eruption
Source: Skin Health Dis. 2023 Jul 13;3(5):e271. doi: 10.1002/ski2.271 (PMC10549868; doi:10.1002/ski2.271)
Supplement: Supplementary file 1 — Supplementary Material [file SKI2-3-e271-s001.docx]

| Supplementary Table 1. Previously reported cases of additive in biologics-related urticaria | | | | | |
| --- | --- | --- | --- | --- | --- |
| Author | Age | Sex | Disease | Biologic agent | Causative additive |
| Kato M, et al. (2019) (1) | 38 | Female | Pustular psoriasis | Adalimumab  Ustekinumab  Ixekizumab  Secukinumab  Brodalumab | Polysorbate20 |
| Pérez-Pérez L, et al. (2011) (2) | 28 | Female | Psoriasis vulgaris | Adalimumab  Ustekinumab | Polysorbate80 |

| Supplementary Table 2. Previously reported cases of biologics-related urticaria | | | | |
| --- | --- | --- | --- | --- |
| Author | Age | Sex | Disease | Biologic agent |
| Our case (2022) | 51 | Female | Palmoplantar pustulosis osteoarthritis | Ixekizumab |
| Rodríguez-Jiménez B, et al. (2009)(3) | 42 | Female | Psoriasis vulgaris | Adalimumab |
| Mallo S, et al. (2007) (4) | 32 | Male | Ankylosing spondylitis | Adalimumab |
| George SJ, et al. (2006) (5) | 41 | Female | Plaque-type psoriasis | Adalimumab |
| Jimenez RB, et al. (2018) (6) | 25 | Female | Psoriasis | Ixekizumab |

Reference

1. Kato M, Oiso N, Uchida S, Yanagihara S, Sano H, Tohda Y, et al. Biologic-induced urticaria due to polysorbate 20. J Dermatol. 2019;46(7):e230-e2.

2. Pérez-Pérez L, García-Gavín J, Piñeiro B, Zulaica A. Biologic-induced urticaria due to polysorbate 80: usefulness of prick test. Br J Dermatol. 2011;164(5):1119-20.

3. Rodríguez-Jiménez B, Domínguez-Ortega J, González-Herrada C, Kindelan-Recarte C, Loribo-Bueno P, Garrido-Peño N. Successful adalimumab desensitization after generalized urticaria and rhinitis. J Investig Allergol Clin Immunol. 2009;19(3):246-7.

4. Mallo S, Santos-Juanes J. Adalimumab-induced urticaria. Actas Dermosifiliogr. 2007;98(7):511-2.

5. George SJ, Anderson HL, Hsu S. Adalimumab-induced urticaria. Dermatol Online J. 2006;12(2):4.

6. Jimenez RB, Vera DG, Rivera-Díaz R, Cortijo-Cascajares S, Ballesteros RM, Pastor M. Successful subcutaneous desensitization in a patient with allergy to ixekizumab. J Allergy Clin Immunol Pract. 2018;6(5):1761-2.
